# Cryopreservation of human kidney organoids

**DOI:** 10.1007/s00018-024-05352-7

**Published:** 2024-07-18

**Authors:** Parham Mashouf, Nahid Tabibzadeh, Shohei Kuraoka, Haruka Oishi, Ryuji Morizane

**Affiliations:** 1 Nephrology Division, Department of Medicine, Massachusetts General Hospital, 149 13th Street, Boston, MA 02129 USA; 2 Harvard Medical School, Boston, MA USA; 3 Harvard Stem Cell Institute (HSCI), Cambridge, MA USA

**Keywords:** Vitrification, Freezing, Kidney, Organoids, Cryopreservation, Dimethyl sulfoxide

## Abstract

Recent advances in stem cell research have led to the creation of organoids, miniature replicas of human organs, offering innovative avenues for studying diseases. Kidney organoids, with their ability to replicate complex renal structures, provide a novel platform for investigating kidney diseases and assessing drug efficacy, albeit hindered by labor-intensive generation and batch variations, highlighting the need for tailored cryopreservation methods to enable widespread utilization. Here, we evaluated cryopreservation strategies for kidney organoids by contrasting slow-freezing and vitrification methods. 118 kidney organoids were categorized into five conditions. Control organoids followed standard culture, while two slow-freezing groups used 10% DMSO (SF1) or commercial freezing media (SF2). Vitrification involved V1 (20% DMSO, 20% Ethylene Glycol with sucrose) and V2 (15% DMSO, 15% Ethylene Glycol). Assessment of viability, functionality, and structural integrity post-thawing revealed notable differences. Vitrification, particularly V1, exhibited superior viability (91% for V1, 26% for V2, 79% for SF1, and 83% for SF2 compared to 99.4% in controls). 3D imaging highlighted distinct nephron segments among groups, emphasizing V1’s efficacy in preserving both podocytes and tubules in kidney organoids. Cisplatin-induced injury revealed a significant reduction in regenerative capacities in organoids cryopreserved by flow-freezing methods, while the V1 method did not show statistical significance compared to the unfrozen controls. This study underscores vitrification, especially with high concentrations of cryoprotectants, as an effective approach for maintaining kidney organoid viability and structure during cryopreservation, offering practical approaches for kidney organoid research.

**Supplementary Information:**

The online version contains supplementary material available at 10.1007/s00018-024-05352-7.

## Introduction

Recent strides in stem cell research have revolutionized the field by the creation of organoids, making the onset of a transformative era propelled by the FDA Modernization Act 2.0 [1–4]. These organoids, intricate miniature replicas of human organs, replicate the cellular diversity and three-dimensional (3D) architecture found in vivo, presenting innovative avenues for investigating diseases in translational medical studies [5–7]. Among these, kidney organoids have garnered considerable attention due to their unique ability to emulate the complex structure and functionality of the renal system. Comprising segmented nephron structures with glomerular and tubular components surrounded by stromal and endothelial elements, these 3D kidney models offer a novel platform for unraveling the mechanisms underlying kidney diseases and for assessing drug candidates in terms of therapeutic efficacy and nephrotoxicity.

The intricate process of creating these sophisticated models involves meticulous experimental procedures, typically encompassing multiple stages of growth factor and small molecule treatment in human pluripotent stem cells. Despite the promising potential of organoids, the labor-intensive nature of their generation and the inherent batch variations in organoid quality pose substantial challenges, limiting widespread utilization across laboratories for diverse biological inquiries aimed at expediting therapeutic development. A potential remedy to this constraint lies in the establishment of cryopreservation methods capable of storing large stocks of organoids. However, cryopreserving organoids, particularly kidney organoids with diverse cell types, demand tailored freezing methods for each cell type to ensure successful preservation without compromising structural and functional integrity.

This study addresses the current gap in cryopreservation techniques for organoids, focusing specifically on kidney organoids. Successful cryopreservation holds the promise of creating an organoid bank that can be shared among research institutions. By overcoming the hurdles associated with labor-intensive organoid generation and ensuring consistency in quality through cryopreservation, the full potential of organoids to drive advancements in medical science can be unlocked. Our inspiration for this endeavor traces back to the pioneering work in cryopreservation, which commenced in 1949 with the intentional and reproducible preservation of fowl sperm [8]. This historical journey, spanning decades, has culminated in the development of a novel freezing method for kidney organoids, as detailed in the present study.

Several inquiries arise regarding the viability of employing standard freezing techniques for these organoids, necessitating exploration into whether specialized preparations are imperative to maintain their viability. In response to these challenges, we endeavored to explore two distinct cryopreservation methodologies—namely, slow freezing and rapid freezing (vitrification)—to safeguard the health and integrity of these intricate structures following the freezing process. We aim to protect the essential elements of kidney organoids, such as proximal tubules, distal tubules, and podocytes. By examining how these techniques affect the preservation of organoid integrity, we seek to provide valuable insights that can inform and enhance the real-world use of kidney organoids in a wide range of scientific and medical applications. In contrast to other types of organoids, kidney organoids exhibit increased depth and intricate structures, necessitating additional measures to uphold their structural integrity and overall health. Employing conventional freezing methods may prove insufficient and potentially detrimental to these complex structures, requiring specialized approaches for preservation. So, one of the most important tips for cryopreservation of kidney organoids is penetration of cryopreservation agents, which should penetrate deeply to preserve some sensitive structures such as podocytes. Prior to embarking on this experiment, various methods were explored and confirmed the essential need for preserving podocytes during cryopreservation. Vitrification, a transformative cryopreservation technique, originally developed in the late 20th century, has already demonstrated its remarkable potential in preserving intricate biological systems, including mammalian embryos and even whole organs [9–15]. Despite its successful application in certain scenarios, the challenge of preserving organ integrity during both cooling and rewarming, especially in larger systems like kidneys, has remained elusive [16–20].

## Slow freezing methods for human kidney organoids

Cellular cryopreservation is commonly accomplished through the implementation of slow freezing techniques, employing the cryoprotectant dimethyl sulfoxide (DMSO). Ice crystal formation stands as a predominant instigator of cellular damage during the cryopreservation process, a challenge effectively addressed by cryoprotectants like DMSO and ethylene glycol, which act to forestall crystallization. The comparative advantages of employing a slow freezing methodology over rapid freezing are noteworthy. This approach demonstrates efficacy in minimizing both crystal formation and cellular dehydration, while concurrently facilitating enhanced penetration of cryoprotectants. This premise is substantiated by antecedent studies showcasing the successful freezing and subsequent thawing of diverse organoids, such as intestinal models [21]. Thus, our initial investigation was designed to evaluate the slow-freezing method for the preservation of kidney organoids. Central to our inquiry was a specific emphasis on ascertaining the viability of maintaining the structural integrity of the multi-layered components within these organoids. The overarching objectives encompassed two fundamental inquiries: firstly, the effectiveness of preserving kidney organoids, encompassing all structures, through the slow freezing method, and secondly, a comparative analysis between the two modes of slow freezing, namely SF1 and SF2, to discern any potential differences in preservation outcomes.

**Fig. 1:**
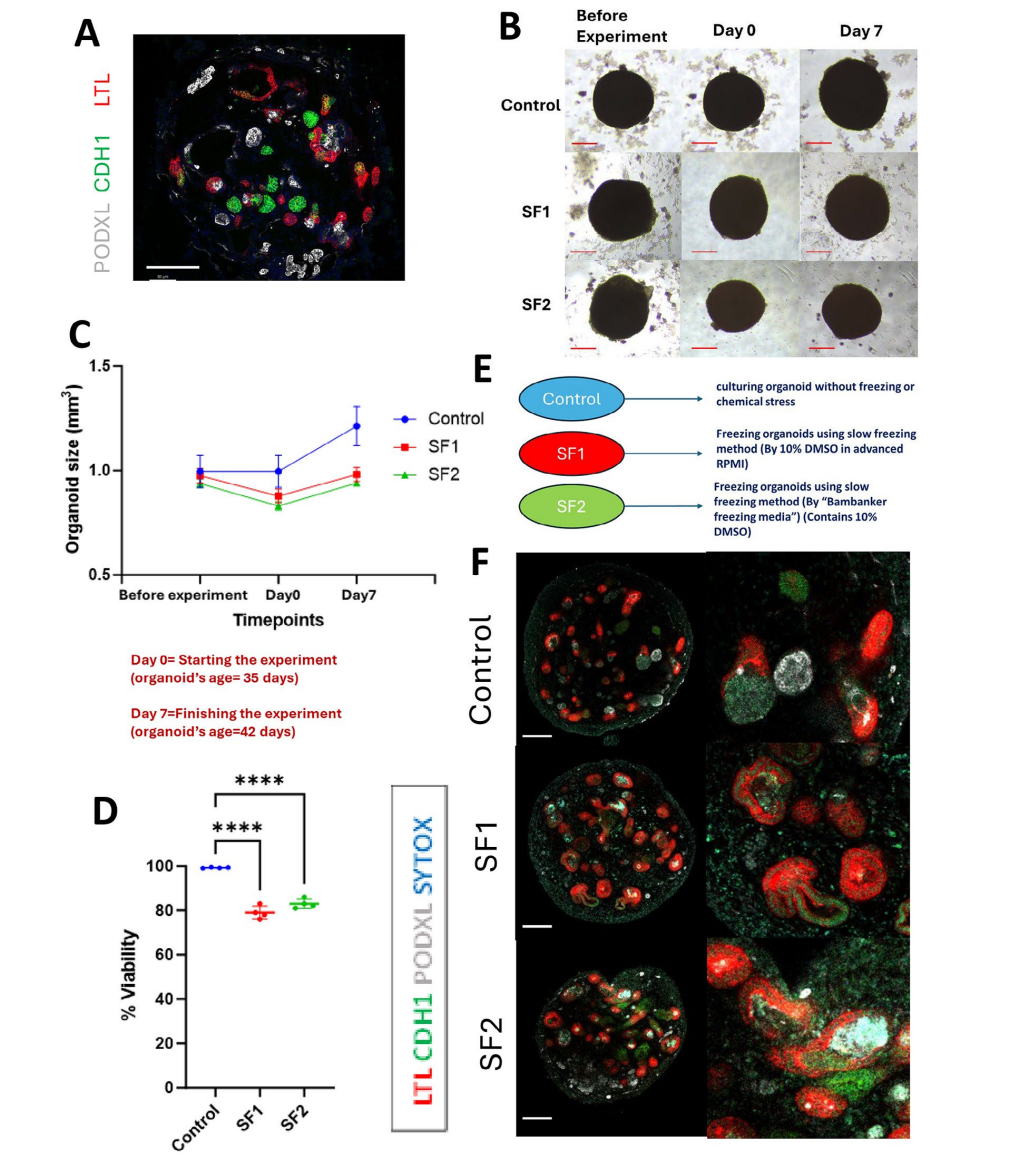
Characterization of kidney organoids following slow freezing: size, viability, and staining. (**A**) An immunostaining representation of a kidney organoid featuring tubular and glomerular structures. Scale bar: 100 μm. (**B**) These images depict the light microscopy views of each condition captured at three distinct time points throughout the experiment: Before freezing (before freezing organoids on day 35 of differentiation), Day 0 (just after thawing), and Day 7 (after 7 days of culture after thawing, equivalent to day 42 of differentiation). Scale bar, 50 μm (bottom). (**C**) A graph depicting kidney organoid sizes across three distinct conditions (Control, SF1, and SF2) at three different time points. The kidney organoids utilized in this study were of the BJFF type, and their age at Day 0 was 35 days. (**D**) A graph representing cell viability across three conditions on Day 0 of thawing, immediately after thawing from the slow freezing conditions. Viability assessment involved the use of trypsin EDTA to disassemble organoid structures into single cells for cell counting with Trypan Blue ****P < 0.0001.(**E**) An exposition of the three conditions within this particular phase of the experiment is warranted. (**F**) Immunostaining images for the assessment of proximal tubules (LTL), loops of Henle, distal tubules, and connecting tubules (CDH1), and podocytes (PODXL) in kidney organoids on day 42 of differentiation with or without slow freezing. Scale bars: 100 μm

Kidney organoids were created by following a meticulously outlined protocol, involving the differentiation of human pluripotent stem cells (hPSCs) through nephron progenitor induction [1]. The resulting intricate three-dimensional nephron structures, encompassing distinct portions from glomeruli to tubular segments [Fig. 1A], featured glomerular structures comprising an outer epithelial layer representing the presumptive Bowman’s capsule and encapsulated podocyte clusters [22]. In pursuit of our objective, we endeavored to safeguard the intricate, multi-layered structures of kidney organoids through the application of the slow freezing method, aiming to ensure the post-freezing health of these organoids in their entirety.

In the pursuit of comprehensively evaluating and elucidating the outcomes of our study, we elected to employ specific methodologies for the preservation and analysis of kidney organoids. The conventional slow freezing technique, designated as SF1, was implemented, utilizing 10% DMSO (Dimethyl sulfoxide) in advanced RPMI with glutamax without equilibration time. Additionally, the commercial freezing media, denoted as SF2, incorporated a cryoprotectant concentration of 10% DMSO and other proprietary components of a commercial freezing product. The assessment of viability was conducted through a commonly used assay for cell viability using Trypan Blue, while structural visualization was facilitated through immunohistochemistry employing LTL (Lotus Tetragonolobus Lectin, proximal tubules), CDH1 (E-Cadherin, loops of Henle and distal nephrons), PODXL (Podocalyxin, podocytes), and SYTOX for nuclei.

Kidney organoids on day 35 of differentiation were cryopreserved by a slow-freezing method utilizing SF1 and SF2. To assess the growth and cellular death, live bright field images were taken before the cryopreservation, and after thawing over one week. While the organoid size was reduced through cryopreservation by approximately 10% in both SF1 and SF2 conditions, a significant increase in size was evident after 7 days of culture in both controls and cryopreserved organoids without apparent dead cells [Fig. 1B and C]. Furthermore, the evaluation of cell viability by Trypan Blue, conducted on Day 0 of thawing through cell dissociation with the trypsin-EDTA, demonstrated a high cell viability of 99.4% in the control samples. However, cell viability for SF1 and SF2 conditions was as low as 79% and 83% respectively. Organoids cryopreserved by the SF1 or SF2 condition exhibited numerous LTL + or CDH1 + tubules, suggesting the preservation of tubular epithelia. However, it was evident that a substantial portion of the tubules had dilated. Importantly, podocyte structures with PODXL staining were rarely observed in organoids cryopreserved by the slow-freezing methods. Qualitatively, SF2 displayed a marginally improved outcome compared to SF1 to preserve podocytes. However, neither medium achieved comprehensive structural preservation. In conclusion, while the conventional slow freezing technique utilizing a standard cryoprotectant proves to be a convenient approach for organoid preservation, its inherent limitation becomes apparent in the inadequacy of preserving podocytes effectively.

## The penetration of cryoprotectants and the formation of ice crystals pose significant challenges when implementing the slow freezing technique

Considering the substantial damage inflicted upon podocytes through slow freezing cryopreservation, we postulated that cryoprotectant penetration was not enough to reach podocyte clusters within Bowman’s capsules, an epithelial layer surrounding podocytes. To enhance the cryoprotectant penetration, we conducted experiments at various equilibration times, a crucial step that allows the cells to adjust to the cryoprotectant solution before freezing. We utilized 10% DMSO as a cryoprotectant at distinct time intervals (0 min, 15 min, 60 min).

**Fig. 2:**
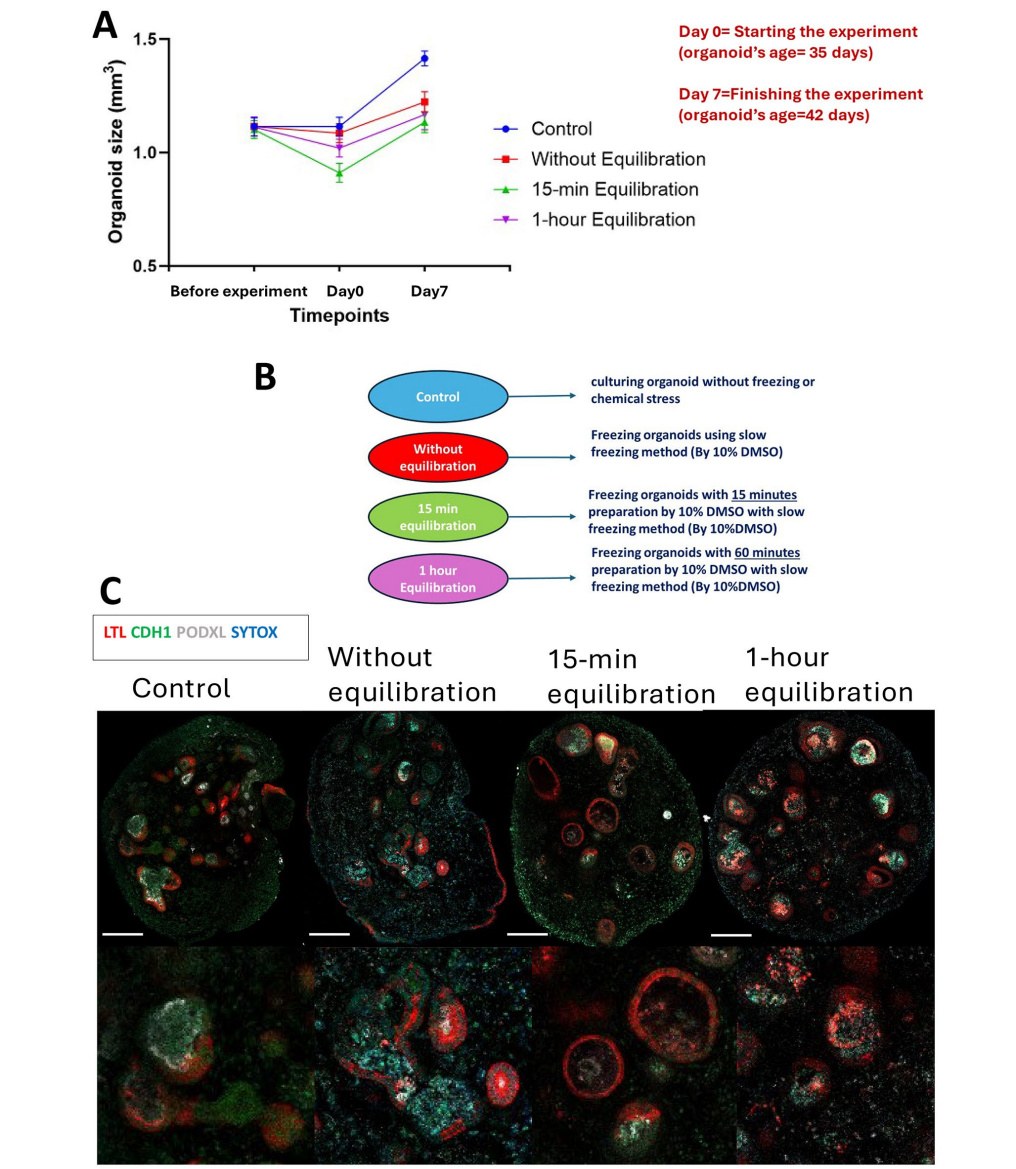
Impact of equilibration duration on kidney organoids during slow freezing: a three-time-point study. (**A**) The comparison of sizes was conducted among all slow-freezing conditions in contrast to the control. Despite utilizing the same freezing method across all conditions, the variable factor was the duration of equilibration. Following freezing, a decrease in size was observed across all subtypes of slow freezing, succeeded by a subsequent normal growth pattern. The kidney organoids utilized in this study were of the BJFF type, and their age at Day 0 was 35 days. (**B**) An elucidation of the three experimental conditions within this segment of the study is shown. (**C**) Immunocytochemistry with sample clearing was employed to examine the features of proximal tubule (LTL), distal tubule (CDH1), and podocyte (PODXL) in all conditions on day 42. The scale bar: 100 μm

In terms of size comparison, the control group exhibited typical growth throughout the experiment. Conversely, consistent with the observations in the slow freezing results previously discussed, all conditions subjected to slow freezing displayed a size reduction immediately following thawing (day 0). Subsequently, they resumed growth after the freezing injury. Notably, there was no statistically significant enhancement in size across any specific sub-condition within the slow freezing protocol. However, it is noteworthy that the percentage of growth after the freezing injury was marginally superior in the condition characterized by a 1-hour equilibration time, although this difference did not achieve statistical significance when compared to other sub-conditions. [Fig. 2A]. The control group exhibited normal kidney organoid structures, displaying intact tubules and healthy cluster-like podocytes. In the slow freezing condition without equilibration, the organoids survived, experienced a reduction in size post-thawing, and subsequently resumed growth under normal incubation conditions. Antibody staining revealed the preservation of only proximal tubules and some distal tubules, while all podocytes incurred damage. For the slow freezing condition with a 15-minute equilibration, a similar post-thawing size reduction was observed, followed by normal growth. Comparative antibody staining indicated greater dilation in proximal tubules compared to the no-equilibration condition, with minimal change in distal tubules. Notably, the damage to podocytes was less pronounced than in the no-equilibration scenario, prompting the exploration of another equilibration timeframe to further assess podocyte preservation. Subsequently, a 1-hour equilibration was implemented, resulting in a post-thaw size reduction similar to previous conditions but with heightened growth rates. However, antibody staining revealed increased damage, particularly in proximal tubules, and the absence of healthy cluster-like podocytes. Despite this extended equilibration, the preservation of podocytes proved elusive. [Fig. 2B, C].

In interpreting the findings, while all subtypes exhibited a return to normal growth post-freezing, exposure to DMSO for over 15 min resulted in significant harm to nearly all structural components. Furthermore, in terms of size expansion, the subtype subjected to a 1-hour equilibration period displayed the greatest increase, albeit concurrently experiencing the highest degree of structural loss. In conclusion, regardless of the specific conditions of slow freezing, the inherent susceptibility of podocytes renders them particularly vulnerable to damage from ice crystals. In essence, although the slow freezing technique presents a convenient method for cell preservation with minimal procedural complexity, its limitations become evident in its inability to effectively preserve cluster-like podocyte structures within kidney organoids.

## Preservation of kidney organoids using vitrification method

The technique of ultra-rapid freezing, commonly referred to as vitrification, has emerged as a prominent method for preserving cells and tissues across various developmental stages. Notably, this process requires mere seconds, preventing the formation of ice crystals and thereby maintaining the freshness of tissues or cells. Recently, vitrification has been employed for preserving entire kidney tissues, yielding remarkable outcomes when transplanted into healthy individuals [23]. Given the multi-layered and intricately structured nature of kidney organoids, which encompass sensitive components such as podocytes and tubules, we have opted to utilize vitrification for their preservation. The objective of this project is to demonstrate the efficacy of vitrification as a freezing method for preserving kidney organoids. Specifically, we aim to investigate the viability of preserving kidney organoids using two distinct vitrification protocols. Our goal is to assess whether this method can maintain all structural components of the organoids in a healthy state, while achieving high cell viability and functionality.

In this study, two subtypes of the vitrification method were employed, each differing slightly in the percentage of cryoprotectant and the duration of certain procedural steps. These subtypes, denoted as V1 and V2, shared the same overarching steps, including equilibration, vitrification, and warming. In V1, equilibration involved a solution of 10% DMSO + 10% Ethylene glycol, while the vitrification solution comprised 20% DMSO, 20% Ethylene glycol, and 0.75 M sucrose. In contrast, V2 utilized equilibration with 7.5% DMSO + 7.5% Ethylene glycol, and a vitrification solution containing 15% DMSO, and 15% Ethylene glycol. Cell viability post-thawing was assessed using trypsin EDTA 0.25%, and the size of organoids was measured after a seven-day thawing period. These particular subtypes were chosen for examination due to their utilization in various medical disciplines, such as gynecology, where they are employed for preserving tissues, akin to the preservation of embryos.

**Fig. 3:**
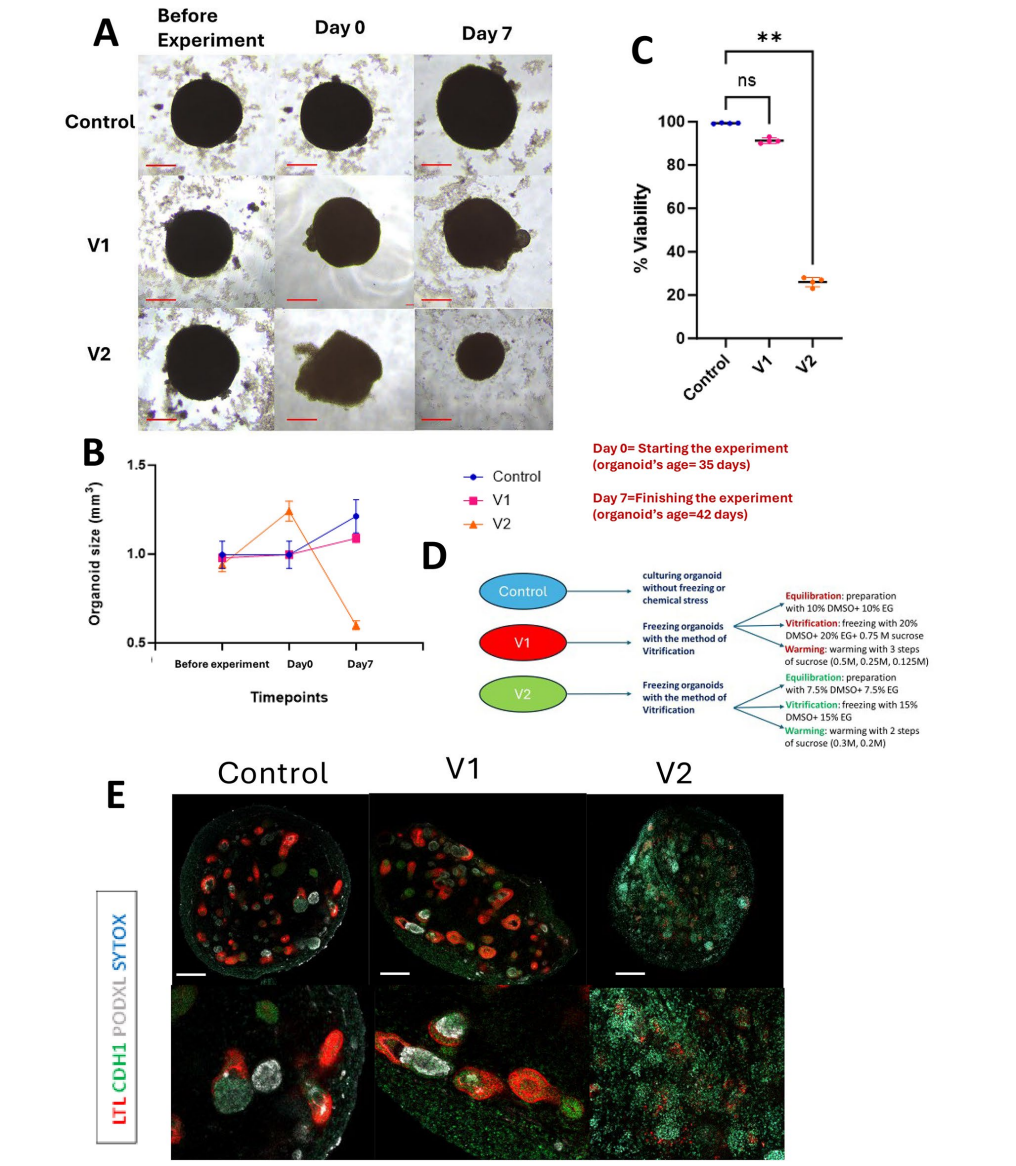
Impact of vitrification on kidney organoids: structural integrity, viability, and size evaluation. (**A**) The bright field images of live organoids in each condition at three distinct time intervals during the course of the experiment: prior to commencement, Day 0 (initiation of the experiment), and Day 7 (conclusion of the experiment). In the context of freezing conditions, Day 0 denotes the stage post-thawing within the experimental setup. Scale bars: 50 μm (bottom). (**B**) A graph depicting kidney organoid sizes across three different conditions (Control, V1, and V2) at three time points: pre-experimentation, the commencement of the experiment (referred to as Day 0, post-thawing for freezing conditions), and Day 7, signifying the termination of the experimental period. (**C**) A graph presenting cell viability across three conditions at Day 0, immediately subsequent to thawing for the vitrification freezing conditions. Viability evaluation entailed the utilization of trypsin EDTA to disassemble organoid structures into individual cells. The average cell viability percentage for the control condition was recorded at 99.4%. The cellular viability in the V1 condition closely resembled that of the control group, registering 91%, with no statistically significant variance observed. In contrast, V2 exhibited substantial cellular damage, with a viability of 26%, demonstrating a significant disparity with ** *P* < 0.01. (**D**) A depiction of the three experimental conditions in this section of the experiment (**E**) Immunocytochemistry for proximal tubule (LTL), distal tubule (CDH1), and podocyte (PODXL) across all conditions on day 42 of differentiation. Scale bars: 100 μm

Kidney organoids at day 35 of differentiation underwent vitrification cryopreservation employing V1 and V2 conditions. While V1 exhibited a minor 2% increase in size, V2 experienced a substantial enlargement, indicative of the extent of damage incurred during freezing. On day 42 of differentiation, following a 7-day experiment, V1 showed an 11% size increase, not significantly deviating from the normal 21% growth observed in the control condition. In contrast, V2 displayed a notable 36% reduction compared to its baseline size, significantly differing from the control, indicating the level of damage sustained. [Fig. 3A, B]. Evaluation of cell viability through Trypan Blue staining on Day 0 post-thawing revealed significant disparities between V2 and the control, while no significant difference was observed between V1 and the control. [Fig. 3C, D]. Organoids cryopreserved with V1 displayed numerous intact proximal and distal tubules with LTL+ and CDH1+ cells, whereas structures in V2 appeared extensively damaged. A crucial aspect of V1 cryopreservation was the preservation of cluster-like podocyte structures, evidenced by PODXL+ signals, whereas V2 exhibited few cells with diminished PODXL + signals, indicative of substantial destruction of podocytes. The control organoids passed all assessments with healthy structures and the highest cell viability compared to these two conditions. [Fig. 3E].

While both methods are commonly employed for cryopreserving cells and tissues, only V1 successfully preserved multi-layer kidney organoids, whereas cryopreservation with V2 proved entirely ineffective for maintaining the integrity of these organoids. In essence, utilizing the suitable vitrification technique, specifically V1, characterized by a higher concentration of cryoprotectants in both the equilibration and vitrification solutions, enables the preservation of all structures within kidney organoids, encompassing the most delicate to the least sensitive components, while ensuring elevated viability. Furthermore, after thawing, organoids exhibit normal growth, suggesting their potential utility in subsequent procedures for diverse applications.

## Tubular dilation: one of the distinctions among freezing methods

The challenges associated with maintaining cellular viability post-freezing prompted researchers to explore various methodologies. However, the fundamental objective of cryopreservation remains the preservation of cellular integrity akin to its pre-freeze state, thereby optimizing efficiency, resource allocation, and cost-effectiveness. This assessment aimed to compare the quantity and size of proximal tubules under different freezing conditions with those of the normal state, thereby identifying a meaningful quantitative parameter indicative of similarity to the control condition (i.e., devoid of freezing-induced stress). Following the preliminary analysis of our investigation, we elected to convert the fluorescence images obtained from the organoids, acquired through confocal microscopy, into quantifiable data. To accomplish this objective, we employed the image analysis software “Imaris”. To optimize the identification of LTL signals and facilitate the generation of structures derived from these signals, the software underwent a machine learning process aimed at eliminating extraneous signals while preserving the primary objects for comparative analysis.

**Fig. 4:**
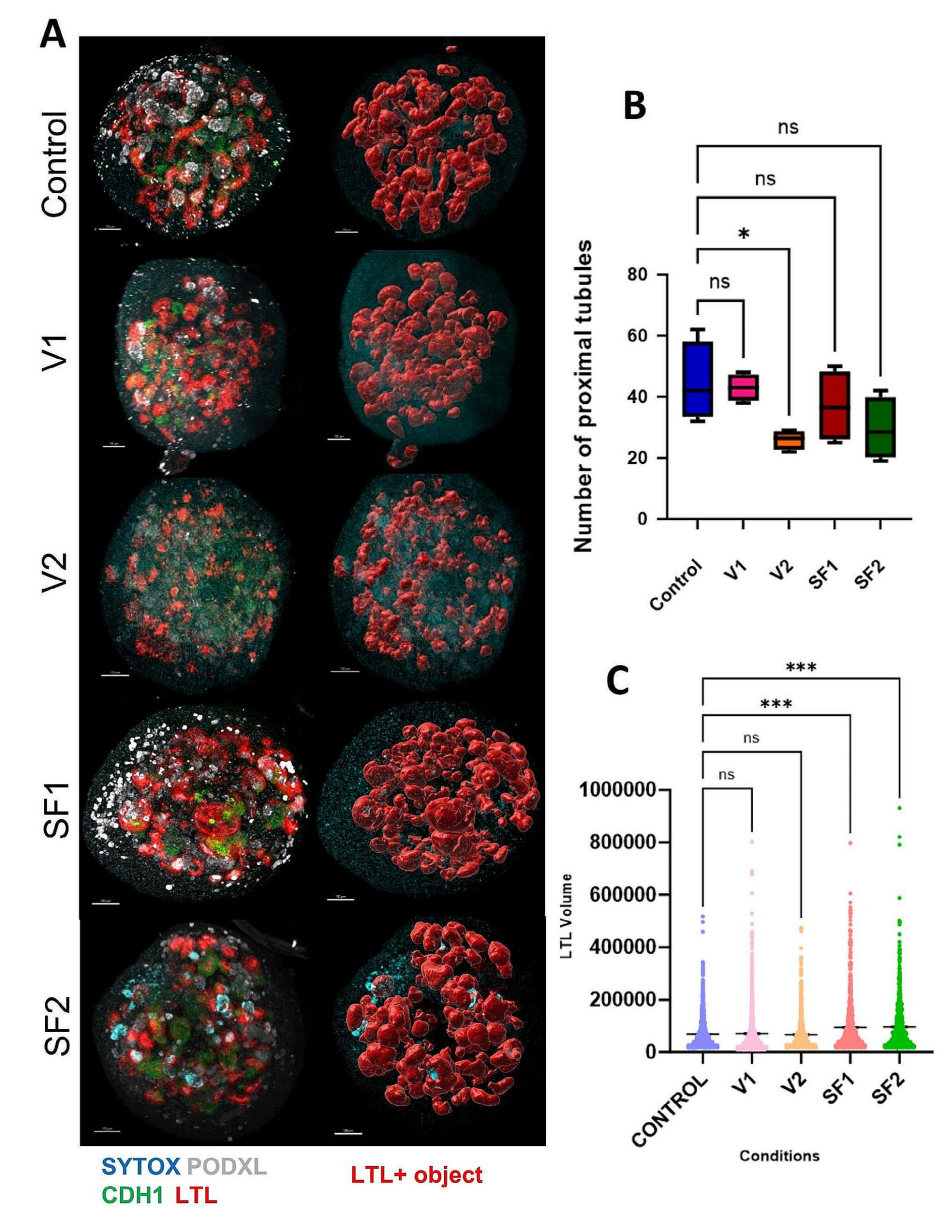
Effect of various freezing conditions on LTL + surface volume and proximal tubule count in kidney organoids. (**A**) Illustrating Images of Machine-Learning-Based Proximal Tubule Structures in Five Experimental Conditions Using Imaris application. (**B**) Comparison chart illustrating proximal tubule counts across multiple experimental conditions. (**C**) Comparison of Volume of Surfaces with LTL-Positive Signals Across All Conditions

The quantitative dataset encompassed the count of proximal tubule structures among the four organoids in each condition. Statistical examination, predominantly employing a t-test, was conducted to evaluate variations in the number of proximal tubule structures across all conditions. The results of this statistical analysis revealed that there were no significant differences (*p*-value > 0.05) in the number of proximal tubule structures among the various conditions except V2. The specific structure counts for each condition were as follows: 45 for the control condition, 43 for V1, 24 for V2, 37 for SF1, and 30 for SF2 [Fig. 4A, B]. Moreover, after the generation of these structures using the Imaris application, a comparative assessment of the surface volumes for proximal tubule structures in all conditions was executed [Fig. 4C]. The resulting graph illustrated that conditions most akin to the control were V1 with no significant difference to the control condition. In the instances of SF1 and SF2, these conditions displayed comparable volumes for proximal structures. The total volume graph also underscored a substantial dissimilarity between these conditions and the control condition, thereby affirming the dilatation of the majority of proximal tubules in both slow-freezing conditions. Following this, the data concerning the surface volume of proximal tubule structures underwent an ANOVA-test analysis. The outcomes of this analysis indicated a significant distinction between the control condition and the slow freezing conditions. Conversely, no significant disparities were observed between the vitrification conditions and the control condition. These results emphasize the efficacy of the appropriate vitrification approach (V1) in achieving outcomes most closely aligned with the control condition.

Upon analyzing the data, it becomes apparent that freezing exerts varying effects on organoids, as evidenced by the findings of this 3D analysis. In both SF1 and SF2, while cryoprotectants contribute to the preservation of structures, freezing-induced tubular dilation occurs in both sub-methods. In contrast, vitrification, owing to the duration of freezing required for organoids, does not result in organoid dilation. This underscores the critical importance of striking a balance between the quantity of cryoprotectant utilized and the duration necessary for freezing the entire organoid to maintain structural integrity and shape. In summary, while slow freezing, encompassing both subtypes, demonstrates efficacy in preserving kidney organoids, it is accompanied by a degree of proximal tubular dilation. In contrast, employing a proper vitrification method offers the advantage of preserving kidney organoids with all their constituent structures and maintaining their original shape intact.

## The existence and number of healthy podocytes: another variance among freezing methods

**Fig. 5:**
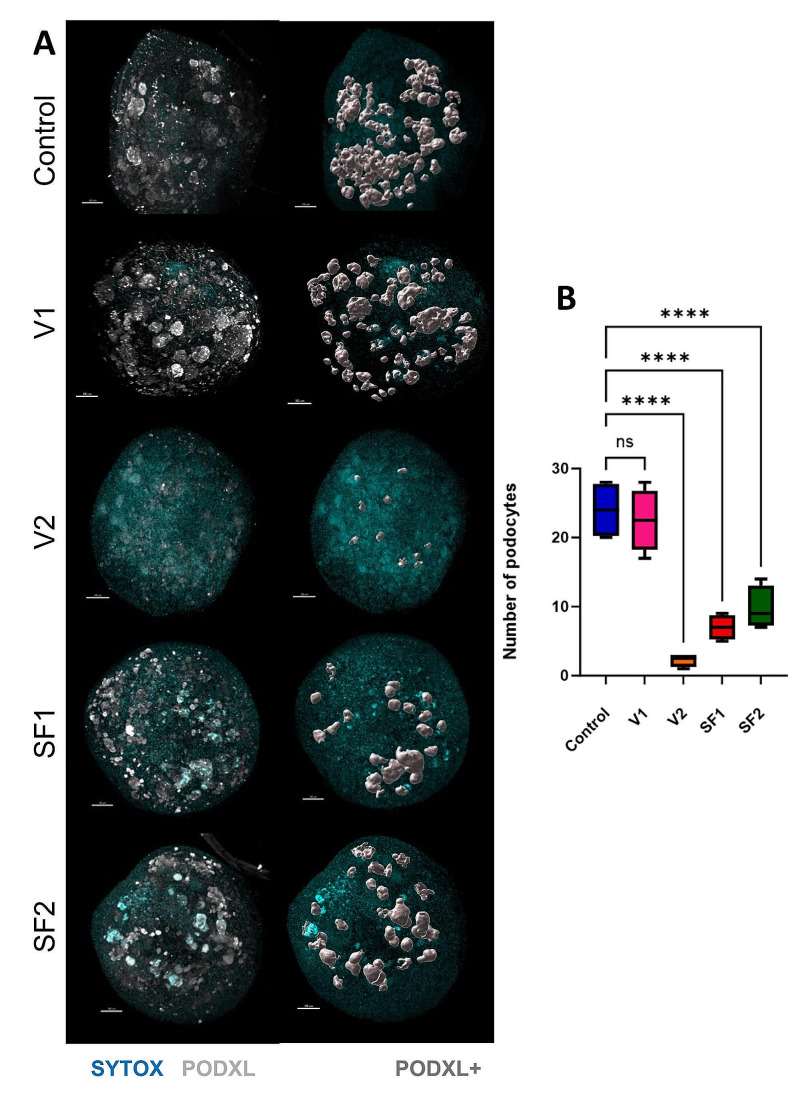
PODXL + surface quantification in kidney organoids: a comparative study using Imaris. (**A**) The characterization of PODXL-positive signal structures in each condition through analysis utilizing the Imaris application. (**B**) Podocyte counts from four replicates per condition following a seven-day incubation and enumeration using the Imaris application

Kidney podocytes are considered terminally differentiated cells originating from nephron progenitor cells (NPCs). Generally, podocytes do not undergo proliferation under normal conditions. Following injury, podocytes typically exhibit a state of limited regenerative activity, characterized by infrequent or absent proliferation phases. A freezing method is deemed valuable when it closely resembles the control group and effectively preserves structures ranging from less to more sensitive ones. The objective of this evaluation is to illustrate the effectiveness of each freezing technique in preserving the highly sensitive component, namely the podocyte, within the complex and multi-layered structure of kidney organoids. To obtain these results, we utilized the “Imaris”. This facilitated the extraction of quantitative data from qualitative images obtained through confocal microscopy of kidney organoids, which had been previously stained via whole-mount staining following tissue clearing procedures. [Fig. 5A]

The outcomes were consistent with our anticipations, as the control group displayed robust growth, amounting to an average count of 24 podocyte clusters across four organoids. In line with the control group, the V1 condition exhibited a profusion of impeccably preserved podocyte structures, notable for their sleek surfaces, resulting in an average count of 22 podocyte clusters among four organoids. In contrast, in the V2 condition, the examination exposed profound structural impairment, resulting in a substantial reduction in the average podocyte cluster count to a mere 2. This result is particularly commendable, given the innate fragility of podocytes. The slow-freezing cryopreservation method in this experiment faced significant difficulties in preserving the vitality of podocytes. These conditions grappled with the task of conserving the majority of podocytes, with podocyte counts of 7 and 10 for SF1 and SF2, respectively. [Fig. 5B]

The statistical examination of these documented podocyte quantities highlighted a notable contrast between the control condition and almost all other experimental conditions. However, the V1 method stood out as an exception, demonstrating no statistically significant difference in podocyte counts when compared to the control group. This result underscores its capability to conserve nearly all podocytes, mirroring its effectiveness in safeguarding other components of the organoids. In conclusion, it appears that the critical factors influencing the preservation of podocytes’ health and structure are the dosage of cryoprotectant and the freezing methodology employed. Optimal preservation of podocytes, characterized by both a sufficient quantity and healthy structure, is achievable solely through the appropriate application of the vitrification method.

## Assessing long-term viability and functionality of post-freezing organoids: utilizing cisplatin toxicity testing

Mechanical stress can induce prolonged injury within replicates, manifesting over time. Stress resulting from freezing conditions has the potential to harm cells both acutely and chronically. While kidney organoids may exhibit short-term tolerance to such stress, the true impact of freezing methods may only become apparent with the passage of time and/or under stress conditions. The primary objective of determining the optimal freezing method is to preserve organoids effectively for subsequent reuse in genuine experimental conditions. This evaluation seeks to address the fundamental question: Can kidney organoids be successfully reused following freezing, and subsequently employed in real experimental settings? To assess the viability and regenerative capacity of kidney organoids post-freezing, a cytotoxicity assay with cisplatin was conducted following Gupta et al.‘s protocol [7, 24]. Organoids were exposed to a 5 μM cisplatin solution for 24 h before replacing them with fresh media. Three replicates per experimental condition were utilized, with a total of six samples for control groups (positive and negative). Media replacements occurred every 48 h, with antibody staining carried out seven days after the commencement of this experiment. Samples were fixed with 4% paraformaldehyde, rinsed with PBS, treated with sucrose, frozen, and sectioned for antibody analysis employing six different antibodies.

**Fig. 6:**
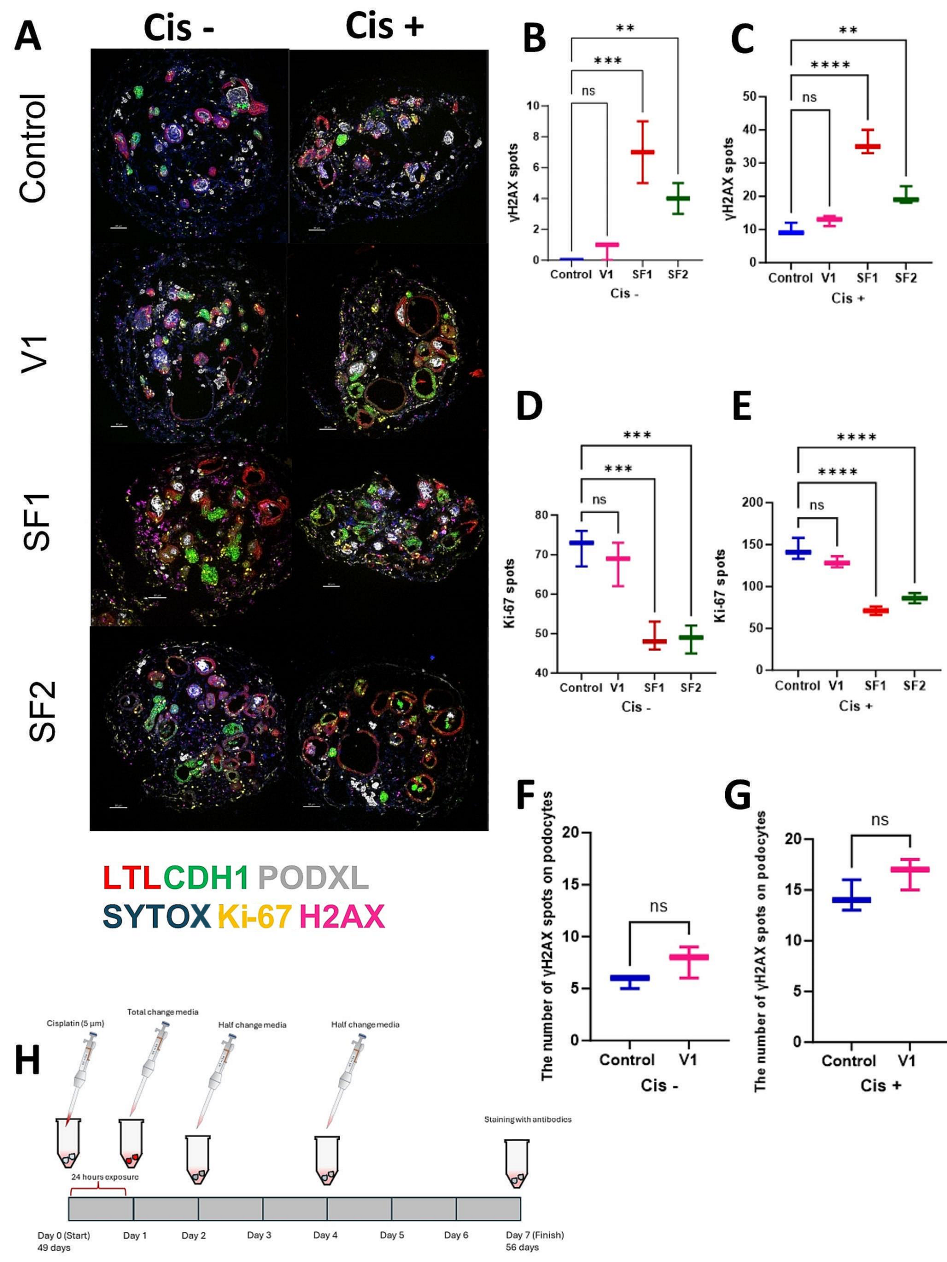
Structural analysis and marker expression after cisplatin toxicity among different conditions. (**A**) Assessing structural integrity, regeneration levels, and damage extent in each condition through antibody staining analysis. (**B**), (**C**), (**D**), (**E**) Quantifying the number of Ki-67 and γH2AX positive spots across replicates serves as a means to assess the degree of regeneration and damage following freezing in comparison to the control condition. (**F**), (**G**) Comparing the number of gH2AX spots, an injury marker, between two conditions with healthy podocytes exposed to cisplatin and without cisplatin. (**H**) The illustrative depiction of the procedural methodology utilized in the experimental assessment of toxicity induced by cisplatin at a concentration of 5 μM

Under typical conditions, our batch of six replicates was evenly split, with three subjected to cisplatin exposure (the positive control) and the remaining three kept free from cisplatin (the negative control). As anticipated, the negative control group exhibited robust signals for LTL, CDH1, PODXL, and Ki-67, minimum γH2AX traces, indicative of double-strand DNA breaks. Conversely, the positive control group showed tubular damage, evident in γH2AX signals, yet notably demonstrated signs of organoid regeneration after cisplatin-induced injury, emphasizing their remarkable recovery capacity. In the V1 condition, findings closely mirrored those of the control. In the negative control, positive signals for LTL, CDH1, and PODXL antibodies were evident. In the V1 an increased count of γH2AX antibody spots compared to the control suggested potential damage due to freezing effects. However, Ki-67 signals in the negative control of V1 indicated potential regeneration post freezing. In the cisplatin samples of V1, expanded proximal tubules with strong LTL signals indicated cisplatin-induced injury, matched by Ki-67 signals signifying subsequent regeneration. The markedly higher count of γH2AX spots in the positive control indicated substantial damage, yet the number of Ki-67 signals in the damaged region emphasized an impressive recovery capacity. However, in the V2 condition, following significant freezing-induced injury, survival beyond seven days was unattainable, with a minimal cell count remaining and a lack of both tubules and podocyte structures for participation in subsequent tests. The SF1 condition demonstrated encouraging outcomes. In the negative control group assessed via section staining, robust LTL signals indicated healthy proximal tubules and CDH1 + signals. The absence of PODXL + cells, indicative of podocyte structures, was attributed to the freezing method employed. While γH2AX expression signified cellular damage in this scenario, the presence of Ki-67 expression indicated the potential for cellular regeneration. In the positive control, tubular damage was observed; these dilated tubules were possibly due to cisplatin injury. Despite remarkable γH2AX signals after cisplatin exposure, the tubules exhibited Ki-67 signals, signifying recovery potential. Notably, apart from damaged podocytes due to freezing, distal tubules appeared most vulnerable, showing the least recovery capacity in this condition. In the SF2 condition, results were consistent with SF1. Strong LTL signals were detected in the negative control, indicating healthy proximal tubules. CDH1+ signals were present but notably lower compared to the control, and podocyte clusters were absent, potentially due to freezing injury. γH2AX signals indicated some degree of damage post freezing, along with Ki-67 signals suggesting cellular proliferation. In the positive control, similar to SF1, some proximal tubules displayed damage and extensive dilation, with fewer tubules compared to the negative control. Ki-67 signals highlighted the recovery potential for proximal tubules. However, akin to SF1, the most vulnerable structure in this test was the distal tubules, exhibiting the least capacity for regeneration. [Fig. 6A–E]

As previously discussed, podocytes, being terminally differentiated cells, generally do not proliferate under normal conditions and exist in an all-or-nothing state, surviving or succumbing to stress. To evaluate the extent of podocyte injury, γH2AX + spots were analyzed using the Imaris application, comparing the optimal freezing method (V1) with control organoids. As anticipated, some injury was observed even in the control group without additional stress. Statistical analysis revealed that although podocyte injury was present in both the V1 and control groups, it was not statistically significant even after exposure to 5 μM cisplatin. Furthermore, numerous healthy clusters were observed in the V1 condition post-cisplatin exposure, comparable to the control group. [Fig. 6F, G]

Our study showcases the resilient nature of kidney organoids, displaying remarkable recovery and regeneration of post cisplatin-induced damage. Notably, V1 exhibited highly promising outcomes, contrasting V2, which couldn’t sustain beyond 7 days and hence was excluded from the experiment. SF1 displayed partial tubular regeneration, particularly in proximal tubules, while SF2 mirrored SF1’s trends, albeit with slightly improved distal tubular regeneration. Regarding γH2AX spot counts indicating damage, V1 demonstrated the most favorable outcome, whereas SF1 exhibited the least favorable results. This research underscores kidney organoids’ resilience to freezing and their ability to recover from injuries, offering valuable insights for potential practical applications.

In conclusion, the superior efficacy of vitrification over slow freezing is evident, as it enables the preservation of kidney organoids with high viability and functionality both in the short and long term. While slow freezing may not perfectly mimic the control condition, it remains a dependable method for certain types of experiments in future research endeavors.

## Quantifying gene expression variations in freezing conditions compared to control through qPCR analysis

**Fig. 7:**
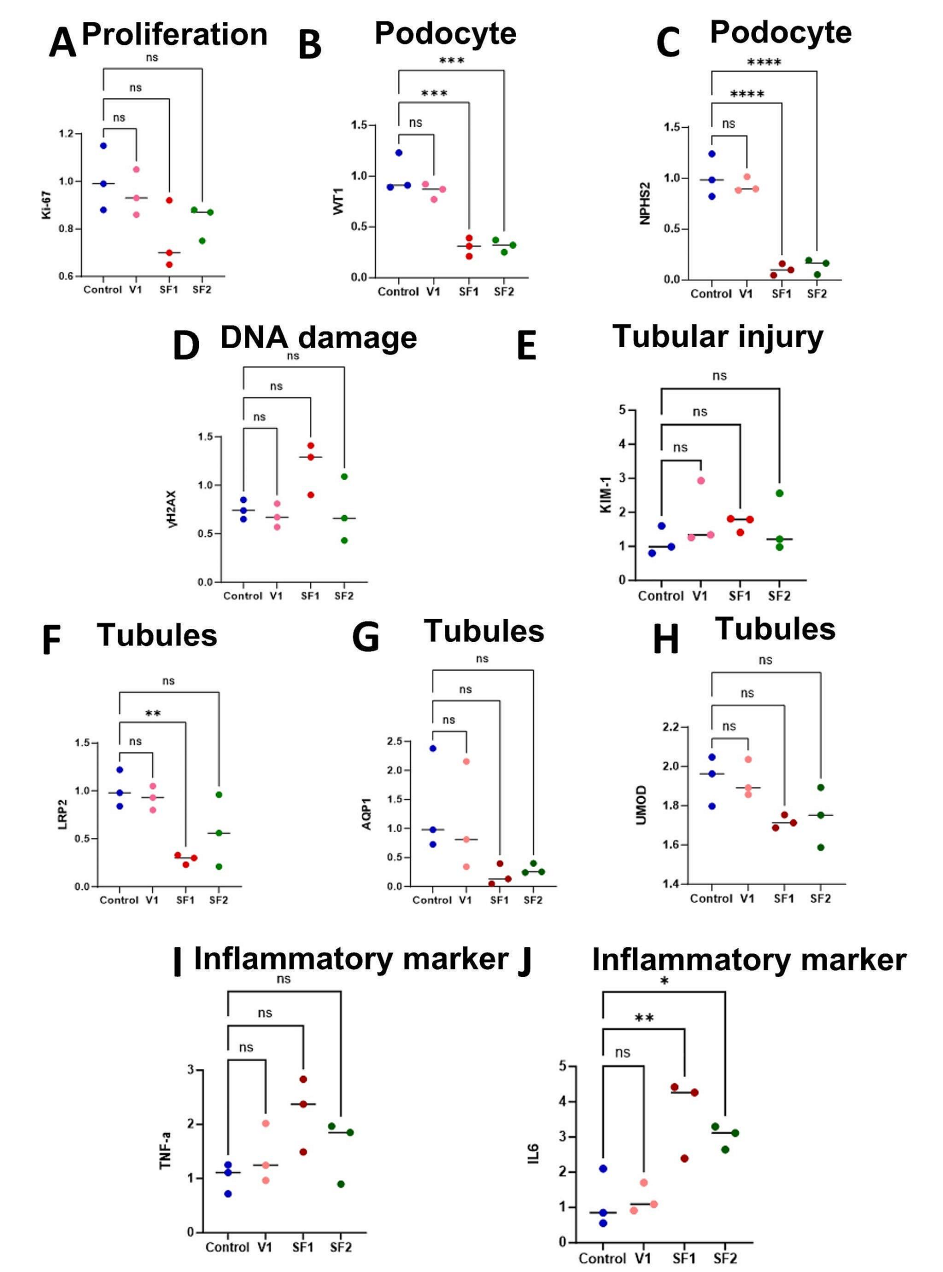
Comparison of marker expression via PCR: structural integrity and injury response. (**A-J**) qPCR for the expression levels of ten distinct genes, specifically Ki-67, WT-1, NPHS2, H2AX, KIM-1, LRP2, AQP1, UMOD, TNF, IL6 across two freezing methods, and their juxtaposition with the control group

As commonly acknowledged, the storage and freezing of cells and tissues have presented significant challenges, with the detrimental effects of freezing often being imperceptible or irreversible upon initial observation. However, the advent of quantitative polymerase chain reaction (qPCR) has provided a rapid means to evaluate the expression of various genes, enabling us to swiftly assess the health and functionality of our tissues or cells. The objective of this assessment is to utilize qPCR to examine the expression levels of specific genes, aiming to assess the extent of damage and the overall health of our organoid genes. After culturing kidney organoids post-thawing for 1 week, at the age of 42 days, we commenced the measurement of the aforementioned genes to assess their expression levels. Following the extraction of RNA and its conversion to cDNA, we quantified the expression levels of ten genes: Kidney injury molecule-1 (KIM-1), Marker of proliferation Ki-67 (Ki-67), Histone H2A.X (H2AX), Wilms’ tumor 1 (WT1), and Low-density lipoprotein receptor-related protein 2 (LRP2), Aquaporin 1 (AQP1), Uromodulin (UMOD), Podocin (NPHS2), Tumor necrosis factor alpha (TNF) and Interleukin 6 (IL6) [Fig. 7]. These measurements were then compared with those obtained from control organoids cultured without exposure to freezing stress.

As anticipated, the control condition, wherein organoids were cultured without stress, exhibited the highest levels of Ki-67 [Fig. 7A], indicative of the normal proliferation process during organoid growth, as well as elevated levels of WT-1 [Fig. 7B] and NPHS2 [Fig. 7C], suggesting a higher percentage of glomeruli compared to other conditions. Furthermore, consistent with the regular cell cycle, they exhibited relatively low levels of H2AX [Fig. 7D] and KIM-1 [Fig. 7E], reflecting the minimum cellular injury. Additionally, the control organoids demonstrated higher levels of LRP2 [Fig. 7F], AQP1 [Fig. 7G] and UMOD [Fig. 7H], markers associated with healthy tubules, particularly proximal tubules and loops of Henle. To verify the inflammatory marker and pathway, we assessed TNF [Fig. 7I] and IL6 [Fig. 7J] levels. The control organoids exhibited the lowest expression, indicating the minimum inflammatory response. In the V1 condition, identified as the appropriate submethod of vitrification, the expression levels of Ki-67 [Fig. 7A] were notably comparable to those of the control group, indicating structural health and ability to regenerate post-freezing stress. Similarly, the levels of WT-1 [Fig. 7B] and NPHS2 [Fig. 7C], prominent markers for podocytes in kidney organoids at 42 days of age, closely mirrored those of the control group, suggesting a substantial presence of healthy glomeruli. Expectedly, the levels of H2AX [Fig. 7D] and KIM-1 [Fig. 7E] were relatively low, suggesting minimal damage. Higher levels of LRP2 [Fig. 7F], AQP1 [Fig. 7G] and UMOD [Fig. 7H] were observed in V1, indicating the overall health of tubules. The inflammatory markers TNF [Fig. 7I] and IL6 [Fig. 7J] were at the lowest levels, comparable to those in the control group. In the V2 condition, identified as another subtype of vitrification, the kidney organoids exhibited a complete failure to survive beyond 7 days. Consequently, they were excluded from the experiment due to extensive structural damage and the absence of viable cells. Within the slow freezing subtypes, SF1 exhibited the lowest levels of Ki-67 [Fig. 7A] among the comparisons, indicating some degree of loss of regenerative capacity. Additionally, WT1 [Fig. 7B] and NPHS2 [Fig. 7C] expression was significantly lower in SF1 compared to the control condition, likely due to podocyte loss during the freezing process using this method. SF1 exhibited the highest levels of H2AX [Fig. 7D] and KIM-1 [Fig. 7E], indicative of more pronounced injury. LRP2 [Fig. 7F], UMOD [Fig. 7G], and AQP1 [Fig. 7H] expression was notably reduced in SF1, suggesting compromised tubular health. In contrast to the control condition, the SF1 condition exhibited the highest levels of inflammatory markers, including TNF [Fig. 7I] and IL6 [Fig. 7J]. In SF2, the results were largely similar to SF1 with slightly improved outcomes. Ki-67[Fig. 7A] demonstrated some degree of proliferation, suggesting cellular health for regeneration. WT1 [Fig. 7B] and NPHS2 [Fig. 7C] levels were markedly lower, as anticipated due to the freezing effects in the slow freezing procedure, indicating the impact of freezing on podocyte loss. H2AX [Fig. 7D] and KIM-1 [Fig. 7E] levels were not significant compared to the control, indicating the effectiveness of slow freezing on organoids, although they were slightly better than SF1. LRP2 [Fig. 7F], AQP1 [Fig. 7G], and UMOD [Fig. 7H] expression was notably reduced due to the impact of the slow freezing process on organoids, although without a significant difference compared to the control group. In terms of inflammatory markers, IL6 [Fig. 7J] showed better results compared to SF1 but remained significant compared to the control. TNF [Fig. 7I] response was slightly lower than SF1 and non-significant compared to the control.

Upon interpreting the results, it becomes apparent that while genes indicative of injury, such as KIM-1 and H2AX, did not show significant differences compared to the control in all conditions, the impact of slow freezing may still prove detrimental to certain structures, notably the glomeruli, as evidenced by the significant difference in WT1 and NPHS2 expression between slow freezing and the control group. In contrast, vitrification did not yield significant differences in WT1 and NPHS2 expression, suggesting the preservation of glomerular health during the vitrification process. Ki-67 expression did not significantly differ between methods, indicating the preservation of most structures in both approaches, with V1 demonstrating the highest Ki-67 levels due to its superior capability for regeneration stemming from healthier structures. As for the LRP2 gene, SF1 exhibited the lowest expression levels, significantly differing from the control, likely due to proximal tubule dedifferentiation and dilation in the slow freezing method. Conversely, V1 displayed expression levels most similar to the control, indicative of preserved tubular integrity during the vitrification procedure. In terms of AQP1 and UMOD expression, although levels were lower in SF1 and SF2 compared to other conditions, there were no significant differences observed. The control and V1 conditions exhibited the lowest TNF and IL6 levels, indicating minimal stress. In contrast, SF1 had the highest levels, suggesting significant stress, with SF2 showing slightly lower but still elevated levels.

In conclusion, the study highlights the effectiveness of a specific vitrification method, V1, in preserving kidney organoid structures and preserving regenerative capacity with minimal damage. V1 demonstrated the least structural damage and the highest regeneration rates, maintaining podocytes similarly to the control group. Additionally, V1 exhibited the lowest inflammatory response after freezing, indicating preserved structural integrity. In contrast, while slow freezing showed notable regeneration potential, it caused significant damage to certain structures such as glomeruli, leading to elevated damage markers. Overall, the optimized vitrification method shows promise for optimal regeneration while minimizing structural damage, emphasizing its superiority in maintaining kidney organoid integrity.

## Materials and methods

### Overview of the experiment

**Fig. 8:**
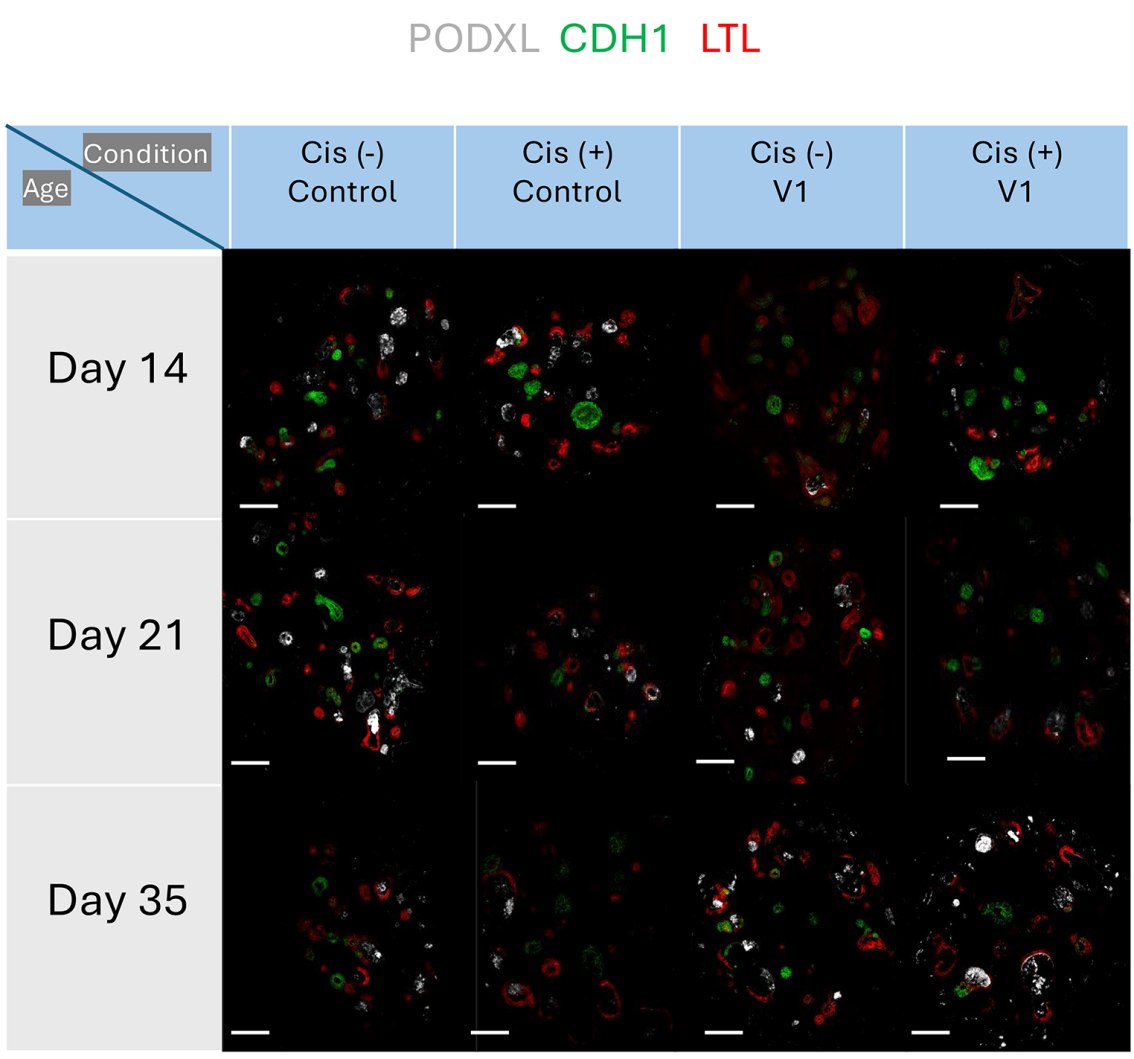
Illustration of the experiment testing the vitrification method on organoids of different ages. Additional experiment testing 5 μM cisplatin toxicity on organoids at 14, 21, and 35 days to demonstrate the effectiveness of vitrification across different ages. After thawing, organoids were cultured until day 49 of differentiation and exposed to cisplatin for 24 h. Samples were harvested on day 56 of differentiation for immunohistochemical assessment (LTL: red, CDH1: green, PODXL: white). For the day 35 kidney organoids, they were thawed from our stocks after 7 weeks to check the effect of storage in the − 150 °C freezer. Scale bars: 80 μm

**Fig. 9:**
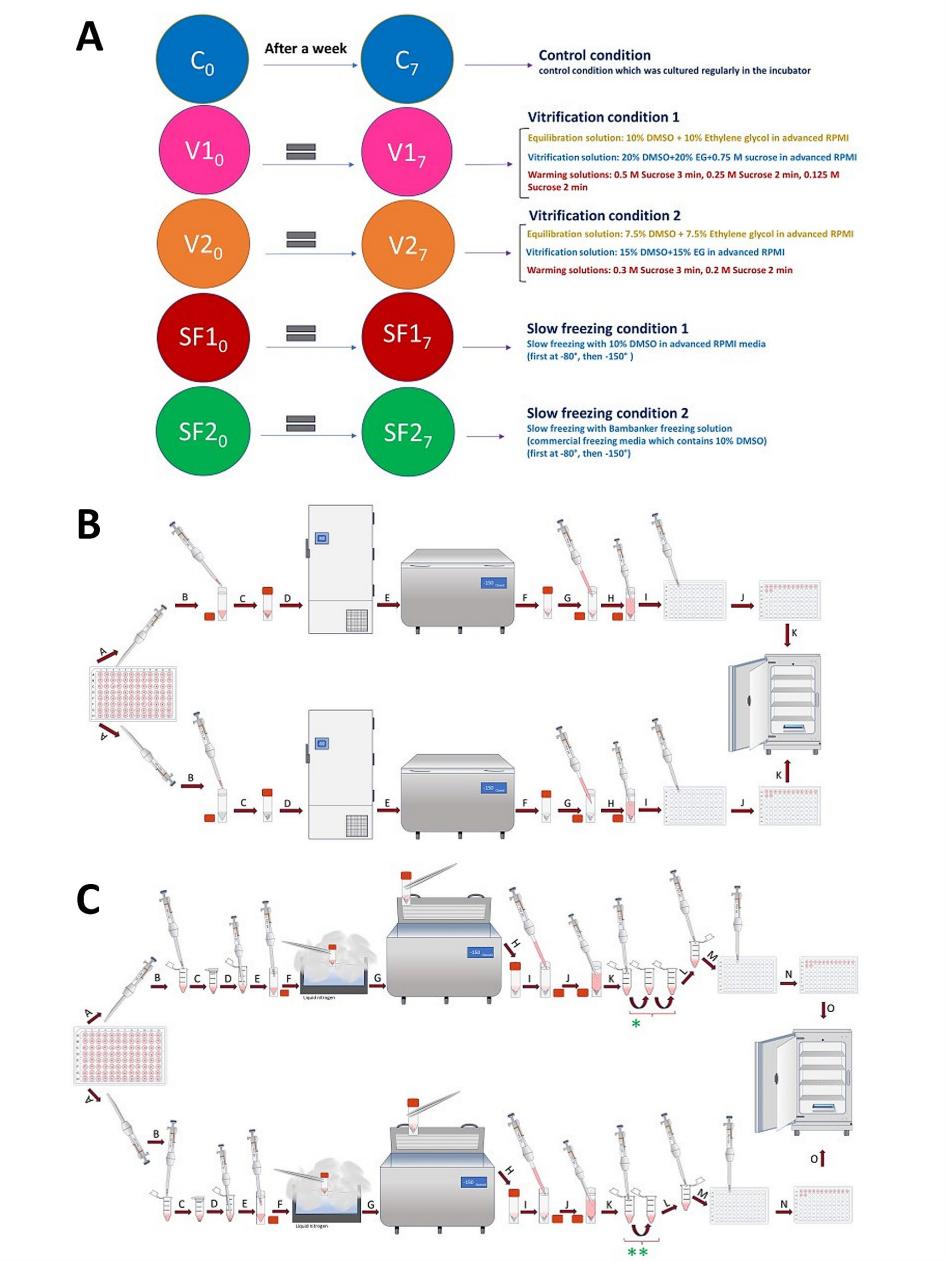
Illustrating the experimental conditions and methodologies utilized for different freezing techniques in this study. (**A**) This study incorporated five distinct experimental conditions, delineating the specifics of each component. All replicates originated from human stem cells, with the kidney organoids being of BJFF type. (**B**) In-depth delineation of slow freezing techniques. The upper section pertains to Slow Freezing number one (SF1), while the lower section pertains to Slow Freezing number two (SF2). All procedural steps remained identical, barring the variation in freezing solutions utilized in this experimental setup. Providing comprehensive elaboration on the sequential steps involved: A- Extracting four organoids using the pipette at its lowest power to minimize media volume. B- Transferring replicates into cryotubes containing 200 μl of freezing media; the upper cryotube (SF1) contains 10% DMSO and advanced RPMI with glutamax, while the lower one (SF2) contains commercial freezing media (Bambanker). C- Ensuring the proper closure of cryotube caps. D- Placing cryotubes in a -80 degree freezer for 24 h. E- Moving cryotubes to a -150-degree freezer for an additional 6 days. F - Retrieving cryotubes after 7 days of freezing for thawing. G - Adding 1 milliliter of fresh room-temperature media for thawing. H - Gently extract replicates using the pipette at the lowest possible power. I - Transferring them into new 96-well plates. J - Moving all 14 replicates of each condition onto the same plate. K - Placing plates into the incubator for regular growth. (**C**) Detailed explanation of ultra-rapid freezing (Vitrification) techniques. The upper segment denotes Vitrification number one (V1), while the lower segment pertains to Vitrification number two (V2). All steps within the process remained consistent, differing solely in the equilibration, vitrification, and warming solutions applied in this experimental setup. A-Extracting four organoids using the pipette at minimal power to minimize media volume. B-Transferring replicates into Eppendorf tubes containing 200 μl of equilibration solution. The upper tube (ES1) comprises 10% DMSO, 10% Ethylene glycol in advanced RPMI with glutamax, and the lower tube (ES2) holds 7.5% DMSO, 7.5% Ethylene glycol in advanced RPMI with glutamax. C-Immerse the organoids into an equilibration solution at room temperature, with exposure times averaging 8–15 min to allow penetration of cryopreserved agents into the kidney organoids. D-Removing replicates to transfer them into specific vitrification solutions. E- Placing replicates into the vitrification solutions; each containing 200 μl. Vitrification solution 1 (VS1) comprises 20% DMSO, 20% Ethylene glycol in advanced RPMI with glutamax, while Vitrification solution 2 (VS2) includes 15% DMSO, 15% Ethylene glycol in advanced RPMI with glutamax. Exposure time should not exceed 30 s due to high toxicity; this process took less than 10 s in this experiment. F- Directly exposing closed-cap cryotubes to liquid nitrogen for 10 s. G- Placing cryotubes directly into the − 150-degree freezer for 7 days. H- Retrieving cryotubes after 7 days of freezing. I- Adding 1 milliliter of fresh room-temperature RPMI media for thawing. J- Removing replicates for multi-step warming solutions. K- Placing organoids into specific warming solutions; each containing 200 μl. *- V1 warming involves 3 steps: 0.5 M sucrose in advanced RPMI with glutamax for 3 min, followed by 0.25 M sucrose for 2 min, and lastly, 0.125 M sucrose for 2 min. **- V2 warming entails 2 steps: 0.3 M sucrose for 3 min then 0.2 M sucrose for 2 min in advanced RPMI with glutamax. L- Retrieving replicates for placement into new 96-well plates. M- Transferring them into the new 96-well plate. N- Moving all 14 organoids of each condition into the same plate. O- Placing the plates into the incubator for regular growth

In this research, a precise and thoughtful arrangement of five unique experimental conditions was established, each centered on BJFF human organoids, collectively involving 118 replicates. Notably, the inception of the project took place on day 35, representing a significant juncture in their development. To evaluate the effect of the optimal freezing method, vitrification, on kidney organoids of various ages and to determine its applicability across different developmental stages, a trial was conducted using organoids at day 14 and day 21 (in addition to the primary organoids on day 35) [Fig. 8]. Vitrification was found to be effective across all ages tested. Day 35 kidney organoids were chosen in this study because organoid transporter activities become more matured at the late stage around day 42 ∼ 49 for cisplatin toxicity test. To test freezing methods, organoids were cryopreserved by 5 conditions structured as follows: one condition served as the control group, two conditions were designated for the slow freezing method, and the remaining two conditions were designated for vitrification [Fig. 9A].

### Control group

Among these five conditions, one functioned as the control group and was exposed to conventional incubator culture conditions. In this particular group, a well-considered approach to media replacement was implemented. Every two days, the media in each replicate was renewed, with each replacement amounting to half of the total media volume. This systematic procedure was employed to guarantee the preservation of an ideal culture environment.

### Slow-freezing conditions

Two conditions were designated for slow-freezing processes [Fig. 9B], labeled as SF1 and SF2. In SF1, the organoids encountered a freezing solution composed of 10% Dimethyl Sulfoxide (DMSO) in advanced Roswell Park Memorial Institute (RPMI) culture media, enriched with Glutamax. Meanwhile, SF2 involved the use of a commercial freezing medium known as “Bambanker”.

In both slow-freezing conditions, 200 μl of freezing medium were loaded into each cryotube, with two replicates for redundancy. The slow-freezing process commenced with an initial storage phase in a -80 °C freezer, followed by a transfer to a -150 °C freezer, resulting in a total freezing period of seven days. Subsequently, for both slow-freezing conditions, the thawing procedure consisted of adding one milliliter of fresh media at room temperature to minimize any dilution of the freezing medium, followed by gentle pipetting steps.

Following this, a comprehensive washing process was conducted using an extra milliliter of RPMI at room temperature. Subsequently, the organoids were retrieved using wide bore tips, reducing the media volume, and then re-suspended in 96-well plates. Each well contained 200 μl of fresh RPMI, pre-warmed and enriched with Glutamax.

Similar to the control group, media replenishment in these conditions involved the replacement of half of the media every two days. Following a seven-day period, all the replicates from these conditions were subjected to a series of tests to evaluate their responses and viability.

### Vitrification conditions

Two more experimental conditions were specifically assigned to the application of vitrification techniques [Fig. 9C]. These conditions were differentiated by the concentrations of cryoprotectants (CPAs) employed. To demonstrate these conditions, we employed two well-established methods commonly used for cryopreservation of tissues and cells, particularly in the field of gynecology (specifically for human blastocytes), which are widely recognized and utilized [27, 28]. In each vitrification condition, a series of three stages was meticulously carried out, encompassing equilibration (aimed at enabling CPA penetration into the organoid structures), vitrification, and subsequent warming solutions.

Following a specified duration in the equilibration solution, the organoids were moved to the vitrification solution, which featured higher CPA concentrations. After this step, the organoids were rapidly immersed in liquid nitrogen for a very brief duration, not exceeding 30 s, to ensure vitrification. Thawing procedures differed between the two vitrification conditions and involved the use of varying sucrose dilutions. The organoids were then placed in 96-well plates containing pre-warmed advanced RPMI culture media with Glutamax, making them ready for further use.

In the initial vitrification condition, denoted as V1 or Vitrification number 1, the equilibration solution consisted of a combination of 10% DMSO and 10% Ethylene Glycol, as per the protocol adapted from the study by Michelle Lane et al. [27]. The organoids were subjected to this equilibration solution for a duration ranging from 8 to 15 min at room temperature, and exposure time varies based on the replicate size.

After the equilibration phase, all replicates were expeditiously moved into cryotubes filled with 200 μl of vitrification solution. This solution consisted of 20% DMSO, 20% Ethylene Glycol, and 0.75 M sucrose. It’s important to highlight that the transfer to the vitrification solution and the subsequent steps for cryopreservation were completed swiftly, taking no more than 30 s, with most lasting less than 10 s.

The cryotubes were promptly submerged into liquid nitrogen to commence the vitrification procedure, followed by placement in a -150 °C freezer. It’s noteworthy that each cryotube contained two replicates, allowing for a thorough evaluation.

After a meticulously defined 7-day vitrification period, the thawing process was carried out with precision. It involved the use of 1 milliliter of fresh media at room temperature, primarily aimed at decreasing the ice blocker concentration. Following this, a three-step warming technique was applied to ensure the gradual recovery of the organoids.

At the outset, the organoids were moved to microtubes filled with 200-microliter volumes of 0.5 M sucrose solution in advanced RPMI for a 3-minute interval. Subsequently, they were relocated to another tube with 200 μl of 0.25 M sucrose solution in advanced RPMI, undergoing an equivalent 3-minute duration. Finally, the organoids were introduced to a tube containing 200 μl of 0.125 M sucrose solution for 2 min, effectively completing the gradual warming process.

After these thorough processes, the organoids were relocated to 96-well plates, and, in adherence to the prescribed protocol, half of the culture media was renewed every two days, guaranteeing their continued viability and growth during the post-thaw culture phase.

In the second vitrification condition, labeled as V2 or Vitrification number 2, the equilibration solution consisted of 7.5% DMSO and 7.5% Ethylene Glycol, following the protocol derived from the research conducted by Tetsunori Mukaida et al. [28]. The organoids were immersed in this equilibration solution for a specific period, which varied based on the size of the replicates and typically ranged from 8 to 15 min at room temperature. Following the equilibration step, the organoids were transferred to individual cryotubes, each containing 200 μl of the vitrification solution.

The vitrification solution in this case contained a mixture of 15% DMSO and 15% Ethylene Glycol, both suspended in advanced RPMI with Glutamax. Similar to the procedure in the first vitrification condition, the transition from equilibration to vitrification was executed rapidly, taking no more than 30 s (with an actual duration of less than 10 s). Subsequently, the cryotubes, each accommodating the organoid replicates, were immediately submerged in liquid nitrogen, marking the initiation of the vitrification process. Following this step, the cryotubes were transferred to a -150 °C freezer and kept there for a precisely defined 7-day duration.

After the completion of the 7-day vitrification period, the thawing process was carried out meticulously. To initiate thawing and reduce the concentration of ice blockers, 1 milliliter of fresh RPMI at room temperature was utilized. The subsequent warming process involved a two-step method. Firstly, the organoids were transferred to microtubes containing 200 μl of a 0.3 M sucrose solution dissolved in fresh RPMI at room temperature, and this step was maintained for 3 min.

Following this, they were transferred to another tube containing 200 μl of a 0.2 M sucrose solution in fresh RPMI at room temperature for an equivalent duration of 2 min. To conclude the stepwise warming process, the organoids were placed in 96-well plates, with each well containing 200 μl of fresh RPMI pre-warmed to room temperature. Just like in all the other conditions, the standard protocol entailed the regular replacement of half the media every two days, ensuring the continual well-being and growth of the organoids during the post-thaw culture period.

### Cell viability evaluation of the organoids after thawing

Upon the conclusion of a 7-day incubation period (when the organoids had reached an age of 42 days), four replicates were chosen from each experimental condition. This was followed by a delicate washing step involving the use of 500 μl of phosphate-buffered saline (PBS) to remove the media. Subsequently, the organoids were subjected to 300 μl of a 0.25% Trypsin-Ethylenediaminetetraacetic acid (EDTA) solution and housed within incubators for a total duration of 15 min, with an initial 7-minute period followed by an additional 8 min.

During the initial 7-minute period, gentle pipetting was executed to ensure the effective exposure of the organoids to the Trypsin-EDTA solution. At the conclusion of the 15-minute incubation, cold RPMI was introduced to neutralize the Trypsin-EDTA, and additional gentle pipetting was carried out to aid the separation of the organoids into individual cells. The subsequent step entailed the use of a centrifuge machine for 4 min at a force of 300 g. Following centrifugation, the supernatant was precisely withdrawn, and 100 μl of RPMI, pre-warmed, were introduced to the cell pellet.

Thorough mixing was performed to ensure uniformity within the solution. To evaluate cell viability, 10 μl of the solution were mixed with an equal volume of trypan blue, and the quantification of cells was subsequently carried out using a specialized cell counting device.

Following the thawing process for all experimental conditions (excluding the control group), a comprehensive series of assessments was undertaken to examine the structural and functional aspects of the kidney organoids. To establish a benchmark, microscopic examinations were conducted by capturing images of the organoids before the initiation of the testing phase, thereby acquiring data concerning their initial dimensions. Subsequently, a decision was made to standardize the measurements for the sake of convenient comparisons. From the project’s inception on Day 0 (or in the case of freezing conditions, post-thawing), until the culmination of the experiment on Day 7, the dimensions of the replicates were subjected to daily scrutiny employing a light microscope and the Image J software. Upon standardization, meticulous comparisons were drawn across the different experimental conditions.

### Quantitative reverse transcription PCR (qRT-PCR)

Total RNA was extracted from kidney organoid samples utilizing TRIzol (Invitrogen) followed by assessment of concentration and purity using a NanoDrop (Thermo Fisher Scientific). Subsequently, cDNA synthesis was conducted from 400 ng of total RNA employing the High-Capacity cDNA Reverse Transcription kit (Applied Biosystems) as per the manufacturer’s instructions. Quantitative PCR was performed utilizing iTaq SYBR Green Supermix (Bio-Rad) on the QuantStudio3 Real-time PCR system. Relative gene expression levels were determined using the delta-delta CT method, with GAPDH serving as the housekeeping gene. Primer sequences utilized are provided in the Supplemental Table 1.

## Discussion

The cryopreservation of living biological tissues and organs has been a focal point of extensive research and development for an extended period. Initial endeavors encountered obstacles, including the formation of ice crystals and tissue damage resulting from these challenges [16, 18, 23]. However, with the introduction of the Vitrification technique and enhancements in related methods and solutions [24–26], investigations comparing diverse cryopreservation approaches, especially the juxtaposition of slow cooling and vitrification, were carried out on a variety of biological materials. These materials encompassed ovarian cells from both animals and humans, as well as human sperm and embryos. While some of these studies did not unveil substantial disparities between the two methodologies, others showcased the superiority of the vitrification method [17–36]. In a study involving mouse kidneys, Han et al. underscored the merits of the vitrification approach in the cryopreservation of mouse kidneys [37].

In this study, we undertook an extensive investigation into the cryopreservation methods, particularly focusing on vitrification and slow freezing, when applied to human kidney organoids. Our main goal was to evaluate how well these techniques maintain the structural soundness and functionality of these intricate three-dimensional structures, with specific attention given to delicate elements like proximal tubules, distal tubules, and podocytes. The results of this investigation provide valuable insights into the crucial role of cryopreservation methods for kidney organoids. The findings of this research offer valuable knowledge concerning the cryopreservation of kidney organoids and the comparison between two widely used techniques: vitrification and slow freezing. These results carry significant implications for the field of regenerative medicine and the possible utilization of kidney organoids.

Foremost, the study underscored the crucial role of cryopreservation techniques in safeguarding the structural and functional soundness of kidney organoids. The selection of a cryopreservation method can significantly influence the results, and the findings from this research underline the effectiveness of the vitrification method, particularly when adhering to the V1 protocol. Vitrification, a technique known for its success in preserving different biological entities, including embryos, demonstrated remarkable proficiency in preserving the size and structural integrity of kidney organoids [19, 20, 26]. This preservation is essential for their utilization in diverse scientific and medical applications.

On the other hand, slow freezing, specifically in the SF1 condition, encountered difficulties in maintaining the integrity of organoid structures, as evident from a minor size reduction on Day 0. Nevertheless, it’s worth highlighting those slow freezing conditions, encompassing SF1 and SF2, displayed signs of recuperation and growth after this initial size decline. This implies that slow freezing might retain some value but demands additional refinement, particularly in the aspect of conserving delicate elements such as podocytes.

The findings regarding the conservation of podocytes hold exceptional importance. Podocytes, recognized for their susceptibility to cryopreservation, play a pivotal role in the functionality of kidney organoids. Notably, the V1 vitrification method demonstrated its efficacy in safeguarding podocytes, with an average tally of 180 podocytes across four organoids. This outcome closely mirrored that of the control group, underscoring the crucial role of vitrification in preserving these fragile components.

On the other hand, the slow freezing conditions, specifically SF1 and SF2, encountered difficulties in maintaining the integrity of podocytes, with recorded podocyte counts of 30 and 50, respectively. This unequivocally demonstrates structural harm to podocytes in these circumstances, providing additional evidence of vitrification’s superiority in this regard.

The quantitative assessment of proximal tubule structures and their surface volumes further supported the advantages of vitrification, particularly when employing the V1 protocol, in preserving the integrity of kidney organoids. Vitrification under the V1 condition closely mirrored the control group, whereas significant dilatation of proximal tubules was evident in the slow freezing conditions (SF1 and SF2). These results highlight the structural robustness achieved through the correct application of vitrification.

For addressing the limitations of this experiment and outlining future research plans, various features of the kidney organoids, including different tubule markers, inflammation markers, proliferation, and injury markers, were intended to be examined using qPCR in the cisplatin toxicity model. However, detailed analysis of each marker can be achieved through alternative methods such as single-cell RNA sequencing, spatial transcriptomics, and/or immunostaining for various purposes such as podocyte injury, other toxicants, cellular metabolism, and genetic disease models. Although numerous markers across different fields were assessed, prior research indicates that the number and mass of mitochondria are lower in iPSCs compared to their original somatic cells [38, 39]. Hence, future experiments may be warranted to focus on this aspect of organoids post-freezing. As detailed in the methods section, the process of thawing organoids after vitrification involves multiple steps. Future studies may also help to further improve the freezing methods with modification or addition of ingredients, such as various ice blockers, to streamline this process and potentially identify a method to preserve the most sensitive part of organoids, the podocytes, in slow freezing methods. For developmental studies, cryopreservation at earlier stages (e.g. differentiation day 14) may be beneficial. Concerning the duration for reusing kidney organoids post-freezing, they were successfully thawed after being stored at -150 °C for seven weeks in our hands. Long-term experiments would determine maximum duration of storage for successful applications to varied purposes.

To conclude, this study strongly advocates for the use of vitrification, particularly when adhering to the V1 protocol, as the primary choice for preserving kidney organoids through cryopreservation. This method reliably upholds both the structural and functional integrity of these organoids, rendering them exceptionally well-suited for diverse scientific and medical purposes. Additionally, the research highlights the imperative for continued investigation and enhancement of slow freezing techniques to enhance their competitiveness with vitrification.

This study’s importance reaches beyond its immediate scope and holds relevance for the wider realm of regenerative medicine. Organoids, such as kidney organoids, serve as pivotal tools in disease modeling, pharmaceutical testing, and transplantation investigations. By offering a dependable cryopreservation technique for kidney organoids, this research may make a substantial contribution to the progress of medical science, with the potential to enhance patient care and results in the times ahead.

## Electronic Supplementary Material

Below is the link to the electronic supplementary material.

Supplementary Material 1

## Data Availability

Any data that support the findings of this study are included within the article.
