# Supplementary material for: Cryopreservation of human kidney organoids
Source: Cell Mol Life Sci. 2024 Jul 18;81(1):306. doi: 10.1007/s00018-024-05352-7 (PMC11335230; doi:10.1007/s00018-024-05352-7)
Supplement: Supplementary file 1 — Supplementary Material 1 [file 18_2024_5352_MOESM1_ESM.docx]

**Supplemental Table 1. Primer sequences**

| Gene | **Reverse (R) amplification** | **Forward (F) amplification** |
| --- | --- | --- |
| **LRP2** | TGTGATGCAGCCATCGAAC | TGCATTTGGGGAGGTCAGTC |
| **WT1** | GGGTACGAGAGGGATAACCA | TCTCACCAGTGTGCTTCCTG |
| **Ki-67** | GCTTGTCAACTGCGGTTGC | GCCTGCTCGACCCTACAGA |
| **H2AX** | CTCAGCTCTTTCCATGAGGGC | CAGGCCTCCCAGGAGTACTAAG |
| **KIM-1** | AAAGGCATTGGAGGAACAAA | CGACAACGACTGTTCCAATG |
| **GAPDH** | CAATGACCCCTTCATTGACC | GACAAGCTTCCCGTTCTCAG |
| **AQP1** | GAGGAGGTGATGCCTGAGAG | GTCCAGGACAACGTGAAGGT |
| **UMOD** | CGGTCTTCAGGCTGACTTTC | AAACCCATGCCACTTACAGC |
| **NPHS2** | CTGTGGACAGAGACTGAAGGG | AGTGCGGATGATTGCTGC |
| **TNF** | GGTTGAGGGTGTGTGCTGAAGGA | CCTGTGAGGAGGACGAACAT |
| **IL6** | TTCACCAGGCAAGTCTCCTCA | CCTGAACCTTCCAAAGATGGC |
